# Inflammation and Interferon Signatures in Peripheral B-Lymphocytes and Sera of Individuals With Fibromyalgia

**DOI:** 10.3389/fimmu.2022.874490

**Published:** 2022-05-26

**Authors:** Serena Fineschi, Joakim Klar, Kristin Ayoola Gustafsson, Kent Jonsson, Bo Karlsson, Niklas Dahl

**Affiliations:** ^1^ Östhammar Health Care Centre, Östhammar, Sweden; ^2^ Department of Public Health and Caring Sciences, Unit of General Practice, Uppsala University, Uppsala, Sweden; ^3^ Science for Life Laboratory, Genetics and Pathology, Department of Immunology, Uppsala University, Uppsala, Sweden; ^4^ Department of Geriatric and Rehabilitation Medicine, Nyköping Hospital, Nyköping, Sweden

**Keywords:** fibromyalgia, B-lymphocytes, RNA sequencing, interferon signature, inflammation, inflammatory proteins, fibromyalgia score, fatigue

## Abstract

Fibromyalgia (FM) is an idiopathic chronic disease characterized by widespread musculoskeletal pain, hyperalgesia and allodynia, often accompanied by fatigue, cognitive dysfunction and other symptoms. Autoimmunity and neuroinflammatory mechanisms have been suggested to play important roles in the pathophysiology of FM supported by recently identified interferon signatures in affected individuals. However, the contribution of different components in the immune system, such as the B-lymphocytes, in the progression to FM are yet unknown. Furthermore, there is a great need for biomarkers that may improve diagnostics of FM. Herein, we investigated the gene expression profile in peripheral B-cells, as well as a panel of inflammatory serum proteins, in 30 FM patients and 23 healthy matched control individuals. RNA sequence analysis revealed 60 differentially expressed genes when comparing the two groups. The group of FM patients showed increased expression of twenty-five interferon-regulated genes, such as *S100A8* and *S100A9, VCAM, CD163, SERPINA1, ANXA1*, and an increased interferon score. Furthermore, FM was associated with elevated levels of 19 inflammatory serum proteins, such as IL8, AXIN1, SIRT2 and STAMBP, that correlated with the FM severity score. Together, the results shows that FM is associated with an interferon signature in B-cells and increased levels of a set of inflammatory serum proteins. Our findings bring further support for immune activation in the pathogenesis of FM and highlight candidate biomarkers for diagnosis and intervention in the management of FM.

## Introduction

Fibromyalgia (FM) is a chronic disease characterized by widespread pain and hypersensitivity to sensory stimuli associated with multiple symptoms such as muscular stiffness, sleep disturbance, cognitive dysfunction, headache, depression, low urinary symptoms, IBS, dry mouth and eyes (1). Fatigue is a common problem in FM contributing to frequent sick leaves among affected individuals. The prevalence of the disease is estimated to between 0.2 and 6.6% with a significant societal impact caused by a reduced productivity and health-care costs (2). Diagnosis of FM represents a challenge and many cases remain undiagnosed due to the lack of specific biomarkers, subjective self-reported questionnaire and a variable constellation of symptoms that may overlap with other disorders (3–6). Moreover, the symptoms may vary over time with alternating periods of improvement and relapse (7, 8). The physical examination may be normal in affected individuals and FM is usually diagnosed exclusively based on the combination of symptoms and a self-reported pain profile (9–11). Given the difficulties in diagnosing FM and its unknown etiology, there is now an urgent need to clarify the underlying pathogenesis and to develop biological markers for the disease.

Prior studies have suggested inflammation, particularly neuroinflammation, and autoimmunity in the pathogenesis of FM (12, 13). High levels of cytokines and chemokines, in particular TNF-α, IL-6, IL-8, IL-17A, have been detected in serum and cerebrospinal fluid of affected individuals (14–23). The correlation between cytokine levels and pain severity is however unclear, as different studies have reported conflicting data (14, 17, 20). Furthermore, the female predominance, antinuclear antibodies (ANA) in 30% of patients and the association with other autoimmune diseases, such as Sjögren’s syndrome, Hashimoto’s thyroiditis and rheumatoid arthritis, have led to hypotheses on autoimmune mechanisms in the pathogenesis of FM (24). Accordingly, accumulating evidences suggest FM to belong to the large group of multifactorial diseases, requiring a genetic susceptibility in combination with yet unknown environmental factors (25, 26).

A recent analysis of transcriptomes from peripheral blood cells of FM cases has reported on T helper cell derived IL-17 (Th-17) and type I interferon (IFN) signatures, i.e. upregulation of IFN-induced genes, suggesting involvement of dysregulated inflammatory pathways associated with the disease (27). Interestingly, type I IFN signature has been reported in the autoimmune diseases Sjögren´s syndrome and SLE sharing some symptoms with FM such as fatigue and general pain (28). On the other hand, fatigue has been reported as a frequent side effect in 70-100% of patients receiving recombinant IFN (29, 30). However, the hypothesis that fatigue is caused by interferon activation in SLE and primary Sjögren´s syndrome is still controversial with inconsistent findings reported in different studies (31–34). The effects of interferon on pain is also unclear although a recent study showed that type I IFN can act directly on nociceptors to generate pain sensitization and generalized pain during viral infections (35). Therefore, the role of interferon behind major symptoms in FM, such as pain and fatigue, remains to be investigated.

B-cells play a pivotal role in autoimmunity by producing pathogenic autoantibodies and by modulating immune responses *via* production of cytokines, chemokines and INF. Furthermore, activated B cells act as potent antigen-presenting cells that may activate autoreactive T cells. The role of the adaptive immune system has not been thoroughly investigated in FM and studies on B lymphocytes in the disease are limited (36). To our knowledge, no report has yet provided a comprehensive analysis of the B-cell transcriptome in FM with correlations to interferons and clinical parameters. Furthermore, recent analysis of serum proteins in FM patients have suggested increased levels of specific inflammatory components. We therefore performed RNA-sequence analysis of B-lymphocytes from FM patients and controls, complemented by a serum analysis of 92 inflammatory proteins. Our aim was to clarify the role of B cells in the pathophysiology of FM and to identify biomarkers associated with the disease. Here, we report that FM is associated with an increased interferon score in peripheral B-cells as well as elevated levels of a subset of inflammatory serum proteins.

## Materials and Methods

### Patients and Controls

We enrolled 30 patients affected by FM and 24 aged and sex-matched healthy control individuals (mean age patients: 48.8 ± 10.9 years and controls: 50.04 ± 11.5 year) from April 2020 to September 2020. Patients and controls were recruited from Östhammar Health Care Centrum, Gimo Health Care centrum and the Pain Clinic of Nyköping Hospital, Sweden. All study subjects were of Scandinavian descent and the control individuals were matched with FM patients for gender, age, BMI, socioeconomic status and educational level. All patients met the 2009 and 2016 ACR criteria for FM. We applied a strict definition of FM requiring exclusion of other disorders that could generate confounding factors in our study and we enrolled only patients with primary FM. Individuals with chronic inflammatory disorders, autoimmune disorders, malignancy, major organ dysfunction, severe depression, use of corticosteroids or immunosuppressive drugs, and ongoing infection were excluded. None of the participants had HBV, HCV and HIV infection. Clinical parameters of patients and control individuals are summarized in **Table S1**. All participants were asked not to provide blood sample if they had a cold or an ongoing infection. One control (F1) experienced high fever the day after sampling, and she was excluded from further molecular analysis. Other exclusion criteria comprised ANA positivity, IgM-rheumatoid factor positivity, high CRP and anti-cyclic citrullinated peptide (CCP) antibodies in order to avoid an interfering inflammatory response caused by other disorders with an autoimmune etiology. Patients and controls had no ongoing or past SARS-COV2 infection as confirmed by negative PCR test and undetectable antibodies. Vaccination against SARS-COV2 was not available at the time of sampling. Patients and controls underwent physical examination including tender point assessment and all study participants completed a questionnaire to assess the fatigue severity scale (FSS; Swedish version), the widespread pain index (WPI) and the symptom severity scale (SSS), confirming extent and distribution of chronical pain. The FSS comprise **9-items** and each item is scored 1-7 (37) as a measure of the severity of fatigue and its effect on a person’s lifestyle. The FM-score (0-31) was calculated for each patient as the sum of the WPI and SSS to assess the severity of the disease (10, 11). All FM patients reported generalized pain and fatigue, and the physical examination confirmed painful tender points but was otherwise normal. Laboratory analysis of erythrocyte sedimentation rate (ESR) and C-reactive protein (CRP) levels, the differential blood count, a comprehensive metabolic panel (except for glucose in patients with diabetes) and thyroid hormone levels turned out normal in all study participants. Similarly, EKG, blood pressure and heart rate were within normal ranges. Participants with comorbidities had their chronic diseases under control. In the control group, none had generalized pain, although some individuals had localized pain. Only one control individual reported to have frequent fatigue with an FFS score of 13. In the FM group, 30 patients affirmed fatigue with a mean FSS of 50.4 (± 9.4 SD). Interestingly, the item number 2 of the FSS (exercise brings on my fatigue) had a mean of 3.4 (± 1.6 SD) and most of the patients affirmed to feel better from regular and low-intense exercise. All patients confirmed generalized pain and the mean FM-score for the entire group of patients was 21.8 (± 4.2 SD) (**Table S1**). The study was approved by the regional ethic committee of Uppsala (2019-00144). All participants gave written consent and the study has performed according to the principles of the Helsinki declaration.

### B-Lymphocyte Isolation and RNA Preparation

Twenty ml of peripheral whole blood were used for isolation of CD19 positive B-cells using MACSprep Chimerism CD19 microbeads, human (Miltenyi Biotec) and AutoMACS Pro Separator (Miltenyi Biotec) according to the manufacturer´s instruction. The purified cell population contained >95% CD19 positive cells confirmed by flow cytometry (Navios flowcytometer, Beckman Coulter). Total RNA was isolated using Trizol (Invitogen), and treated with DNA-free™ DNase and Removal Reagent (Ambion, Life technologies, CA, USA). The final quantity of RNA obtained from the CD19 positive B-cells varied from 0.1 to 4.7 ug and was quality assessed using Agilent Tape Station 2200 (Agilent, Santa Clara, CA, USA). All RNA samples had RNA integrity number (RIN) values of >8.

### RNA Sequencing

A total of 100 ng of RNA from purified B-cells of each participant (26 patients and 23 controls) was used for sequencing following rRNA depletion and library preparation (TruSeq stranded mRNA library preparation kit, cat# 20020595, Illumina Inc.). Unique dual indexes (cat# 20022371, Illumina Inc.) were used according to the manufacturers’ protocol (#1000000040498). The quality of libraries was evaluated using the Fragment Analyzer from Advanced Analytical (AATI) using the DNF-910 kit and adapter-ligated fragments were quantified by qPCR using the Library quantification kit for Illumina (KAPA Biosystems) on a CFX384Touch (Bio-Rad) prior to cluster generation and sequencing. Sequencing was carried out on an Illumina NovaSeq 6000 instrument (NovaSeq control software v 1.7.0/RTA v3.4.4) according to the manufacturers’ instructions. Demultiplexing and conversion to FASTQ format was performed using the bcl2fastq2 (v2.20.0.422) software, provided by Illumina. Additional statistics on sequencing quality were compiled with an in-house script from the FASTQ-files, RTA and BCL2FASTQ2 output files.

### Data Analysis

Samples were analyzed with the nf-core RNA sequencing pipeline release 1.4.2 (github.com/nf-core/rnaseq). In brief, the pipeline processes raw data from FastQ inputs, aligns the reads, generates counts relative to genes or transcripts and performs extensive quality-control of results. Power estimation by read count and dispersion was performed using the R package RnaSeqSampleSize (Release 3.14) (38). The functions est_count_dispersion and est_power_distribution were used to calculated dispersion in our data set to 0.0852, resulting in a power estimation of 0.896 (n=23; rho=2; repNumber=500) at an FDR set to 0.05. The online version of xCell (xcell.ucsf.edu) was used to evaluate the cell composition of sorted cells from the transcriptomes (39). A set of seven different immune cell signatures were used (40). Heatmap of the xCell results was generated using the xCell heatmap viewer (comphealth.ucsf.edu/app/xcellview). Prior to further analysis, genes with zero reads in more than five samples were filtered out. Subsequently, raw counts were pre-processed and used for differential expression analysis by the R package DESeq2 (v1.30.1). Heatmaps were produced using the R package ComplexHeatmap (v2.6.2) illustrating Z-scores for each gene and clustering was performed on the Euclidean distances between genes and samples. Volcano plot was generated using the R package EnhancedVolcano. We used the ropls package (v1.27.4) to perform Orthogonal Partial Least-Squares (OPLS) between the experimental groups. Gene Set Enrichment Analysis (GSEA) was performed on all genes after ranking them according to the sign(log2Foldchange)*-log10(pvalue) using the R package clusterProfiler (v3.18.1). MSigDB gene set collection was obtained from baderlab.org. Visualization of the GSEA results was done using the R package enrichplot (v1.10.2).

IFN-regulated genes were assigned according to the Interferome V2-0 database. The number of interferon-regulated genes with increased or reduced expression in FM patients vs. controls was evaluated using FISHER exact t-test. Expression values of *IFI27, IFI44L, IFIT1, ISG15, RSAD2*, and *SIGLEC1* were used to calculate an “IFN score” for each individual and according to an established approach to minimize inter-laboratory variability in IFN signature analysis (41). In brief, the average expression and standard deviation of each of the six genes in the control group were used to derive expression scores [(Expression score = (expression in an individual – average expression in controls)/standard deviation in control)] as previously described (42). The sum of expression scores of the six genes were then calculated for each individual. The difference in IFN score between groups was estimated using an unpaired two-sided t-test.

### Detection of Inflammatory Proteins in Sera

A total of 53 sera (30 patients and 23 controls) were analyzed using the Olink Inflammatory panel (Olink Proteomics AB, Uppsala, Sweden) by the analysis service at Olink Proteomics AB, Uppsala, Sweden. Data were expressed as normalized protein expression (NPX) values, Olink Protemics’ arbitrary unit, on a log2 scale. NPX values were acquired by normalizing cq-values against extension control as well as an interplate control and a correction factor. A high NPX value corresponds to a high protein level that can be used for statistical multivariate analysis to express the relative quantification between samples. Data was analyzed using the R-package OlinkAnalyze (GitHub: github.com/Olink-Proteomics/OlinkRPackage/tree/master/OlinkAnalyze). Differences between groups was calculated using t-test adjusted for multiple testing using the Benjamini-Hochberg method. Heatmaps was generated using the R package pheatmap (version 1.0.12) and show the Z-scores for each protein.

## Results

### Differentially Expressed Genes in B-Cells of FM Patients vs. Control Individuals

**Figure 1:**
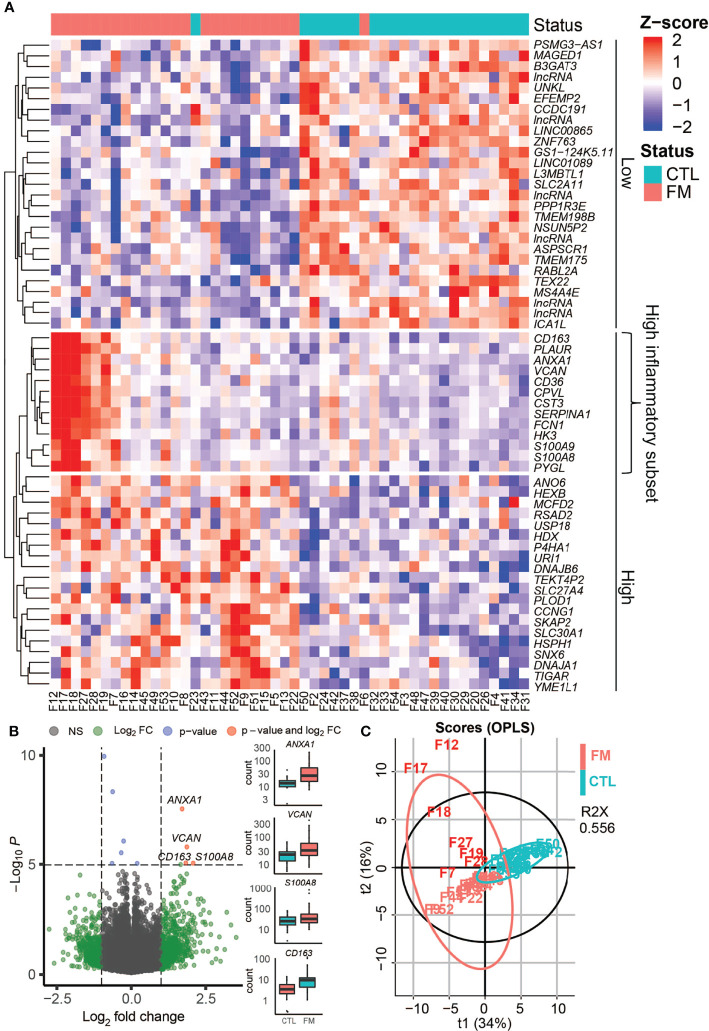
Gene expression profile in B-cells of FM patients compared to controls (CTL). Gene expression profile in B-cells of FM patientes compared to controls (CTL). **(A)** Heat-map shows differentially expressed genes in 25 FM patients vs. 23 control individuals. The genes are grouped if they show a lower expression in FM patients (Low) or a higher expression in FM patients (High). A group of inflammatory genes with a very high expression in seven patients is indicated (High inflammatory subset). **(B)** Volcano plot illustrating differentially expressed genes (green and red dots) in the 25 FM patients vs. 23 control individuals. A marked difference is shown for genes with a strong connection to inflammation and autoimmunity such as *ANXA1, VCAM, CD163, S100A8*. **(C)** OPLS analysis of differentially expressed genes in FM patients (light red) vs. controls (magenta) allowed for a separation of the subset of FM patients with high expression of inflammatory genes (F7, F12, F17, F18, F19, F27, F28; dark red).

We sequenced total RNA in sorted B-cells of 26 patients and 23 healthy, matched control individuals. In order to identify dysregulated genes in patients vs. healthy control individuals we removed transcripts without mapped reads in more than five samples resulting in 19’130 transcripts that remained shared between the FM and control groups. Normalization was applied in order to control for inherent technical variables, such as different sequencing depths (**Figure S1A**). We then performed a Principal Component Analysis (PCA) of all samples in order to examine the overall distribution of RNA-seq data (**Figure S1B**). The analysis of the two first principal components identified one outlier sample (F36). Analysis of the transcriptional signatures using xCell software confirmed a predominantly B-cell derived signature (average xCell-score 0.96 +/- 0.002) in all samples but one (F36). Secondary cell signatures were characteristic for neutrophils (average xCell-score 0.73 +/- 0.03) and dendritic cells (DC; average xCell-score 0.61 +/- 0.03) (**Figure S2**). Importantly, the analysis did not show any macrophage signature (average xCell-score 0.29 +/- 0.06). The outlier sample (F36) transcriptional signature showed similarities to that of neutrophil cells (xCell score 0.88) (**Figure S2**) and, therefore removed in further analysis. When comparing data from the remaining 25 FM patients with 23 controls, we identified 60 differentially expressed genes (adjusted p-value <0.05), of which 33 genes showed increased expression and 27 genes a reduced expression in FM patients when compared to controls (**Figure 1** **;** **Table 1**). We then determined the Euclidean distances between all samples using their gene expression profiles (**Figure 1A**). The cluster of genes with reduced expression (n=27) in FM patients comprised mainly long non-coding RNA (lncRna) and pseudogenes of unknown functions **(** **Table 1**). In contrast, the gene cluster with increased expression in FM consisted to a large extent of IFN-responsive genes (25 out of 33, 76%; p = 0.0002) (**Table 1** **)**. Furthermore, within the cluster of upregulated genes, we identified a distinct group of genes with a markedly increased expression in a subset of seven FM patients (n=7; F7, F12, F17, F18, F19, F27, F28) (**Figure 1A**). The highest expression was observed for the IFN-regulated and proinflammatory genes *S100A8*, *VCAM*, *CD163* and *ANXA1* **(** **Figure 1B** **;** **Table 1** **).** We annotated the group of seven FM patients with this expression profile as a “high inflammatory subset” (**Figure 1A**). In addition, we applied an OPLS regression model to identify class-discriminating genes in FM patients and controls. The computation showed a clear separation between the FM and the control group. Furthermore, within the FM-group, the model identified the subset of seven FM patients with a marked relative increase in expression of proinflammatory genes (**Figure 1C**).

**Table 1:** Differentially expressed genes and transcripts between FM patients and controls.

Gene	base Mean	log2FC	FDR	IFN-regulated
**Upregulated**				25/33
S100A8*	71.3	2.05	0.020	YES
VCAN*	48.7	1.85	0.0082	YES
S100A9*	150.5	1.84	0.029	YES
CD163*	9.5	1.81	0.020	YES
SERPINA1*	86.5	1.70	0.030	YES
ANXA1*	32.1	1.68	0.00042	YES
PLAUR*	10.9	1.67	0.044	YES
CPVL*	37.4	1.63	0.020	YES
HK3*	34.5	1.60	0.030	YES
FCN1*	186.6	1.59	0.048	YES
CD36*	21.8	1.56	0.030	YES
*TEKT4P2*	53.7	1.50	0.024	
CST3*	66.2	1.45	0.037	YES
PYGL*	16.4	1.43	0.049	YES
*USP18*	52.3	0.61	0.029	YES
*HDX*	56.3	0.53	0.034	YES
*RSAD2*	117.5	0.50	0.029	YES
*SLC30A1*	199.0	0.39	0.042	YES
*TIGAR*	224.9	0.32	0.020	
*PLOD1*	342.1	0.27	0.042	YES
*HSPH1*	2078.1	0.23	0.042	
*SLC27A4*	416.9	0.21	0.049	
*CCNG1*	1991.9	0.21	0.024	YES
*P4HA1*	1046.2	0.21	0.029	
*SKAP2*	2309.0	0.20	0.029	YES
*DNAJB6*	2733.1	0.19	0.020	YES
*DNAJA1*	1903.0	0.19	0.029	YES
*ANO6*	1190.2	0.16	0.044	
*HEXB*	891.3	0.15	0.024	YES
*SNX6*	1519.2	0.12	0.008	YES
*URI1*	2001.5	0.12	0.020	
*MCFD2*	1951.9	0.11	0.041	
*YME1L1*	3844.7	0.10	0.049	YES
**Downregulated**				7/27
*lncRNA*	46.2	-0.90	9.28E-06	
*MS4A4E*	14.4	-0.87	0.039	
*LINC00865*	189.3	-0.64	0.020	
*lncRNA*	105.8	-0.62	0.00011	
*Lnc-GPA33-3*	29.0	-0.46	0.018	
*EFEMP2*	160.4	-0.41	0.026	
*lncRNA*	72.0	-0.36	0.020	
*CCDC191*	1752.9	-0.35	0.005	
*TEX22*	82.3	-0.31	0.042	
*ZNF763*	143.5	-0.29	0.021	
*LINC01089*	1341.3	-0.28	0.00017	
*lncRNA*	417.7	-0.26	0.029	
*PSMG3-AS1*	549.5	-0.25	0.041	
*ICA1L*	278.7	-0.22	0.030	
*Lnc-ZNF843-2*	206.5	-0.22	0.029	
*SLC2A11*	1536.6	-0.21	0.029	YES
*MAGED1*	1578.2	-0.21	0.029	YES
*TMEM175*	2896.4	-0.19	0.018	
*UNKL*	2286.7	-0.18	0.018	YES
*ASPSCR1*	1110.6	-0.18	0.034	
*PPP1R3E*	2514.3	-0.18	0.029	YES
*RABL2A*	1107.5	-0.18	0.030	
*TMEM198B*	1411.5	-0.17	0.041	YES
*L3MBTL1*	1091.9	-0.16	0.041	YES
*NSUN5P2*	2665.0	-0.15	0.024	YES
*GS1-124K5.11*	1384.1	-0.15	0.042	
*B3GAT3*	1829.3	-0.14	0.032	

The base mean is the mean of normalized counts of all samples. Log2FC is the log2 fold-change between patient vs control. FDR is the Benjamini–Hochberg adjusted p-values from DESeq2. IFN-regulated genes annotated from the Interferome V2-0 database. *Genes or transcripts showing high expression in a subgroup of patients.

### Enriched Pathways and Increased Interferon Scores in FM Patients vs. Control Individuals

**Figure 2:**
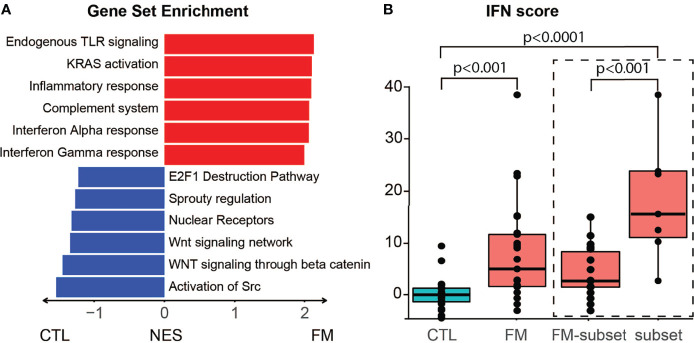
Gene enrichment analysis and IFN score in B-cells of FM patients. **(A)** Differentially expressed genes predict interference with specific pathways. Top upregulated pathways in FM patients comprise TLR signaling, KRAS activation, inflammatory response, complement system, interferon alpha and gamma response. **(B)** The group of 25 FM patients show a higher IFN score when compared to the 23 control individuals (p=4.13x10^-4^). The high inflammatory subset of seven FM patients (subset) show a higher IFN score compared to both controls (p = 4.36x10^-7^) and to the remaining FM patients (FM-subset; p = 5.24x10^-4^).

We then sought to investigate the predicted effects of differentially expressed genes on known functional pathways. Enrichment analysis revealed that the top six terms were associated with inflammation, namely Endogenous TLR signaling, KRAS activation, Inflammatory response, Complement system, Interferon alpha and gamma response (**Figure 2A** **;** **Table S2**). Conversely, reduced activity was found for several regulatory pathways. We then used the expression values of six IFN genes to calculate an IFN-score. Notably, the 25 FM patients showed an increased IFN-score when compared to the group of 23 controls (IFN-score: control = 0.0 ± 3.4; patient = 8.6 ± 10.4; p-value = 4.13x10^-4^). Furthermore, the seven FM patients belonging to the “high inflammatory subset” showed a more marked increase in IFN-score compared to controls (Subset IFN-score = 17.1 ± 12.1; p-value = 4.36x10^-7^; **Figure 2B** **;** **Table S1**).

### Correlation Between Molecular and Clinical Parameters Within the Group of FM Patients

We next wanted to compare the “high inflammatory subset” of seven FM patients, showing the highest expression of pro-inflammatory genes, with respect to the remaining 18 FM patients. We identified in total 54 differentially expressed genes, 41 with a higher expression and 13 with a lower expression, in the seven FM patients (adjusted p-value < 0.05 and log2(FC) ≤ -0.5 and ≥2.5 (**Figure S3A** **;** **Table S3** **)**. Enrichment analysis revealed increased activity for “TNF-alpha signaling *via* NF-kB”, “Inflammatory response” and “Endogenous TLR signaling” (**Figure S3B** **;** **Table S4**). Notably, the “high inflammatory subset” of FM patients showed an increased IFN signature compared to the remaining FM patients (17.1 ± 12.1 vs 1.7 ± 3.8; p-value = 5.24x10^-4^; **Figure 2B** **;** **Table S1**). The seven patients of the “high inflammatory subset” were therefore called for additional examinations and interviews in search for any correlations between clinical manifestations and the transcriptional profile. Analysis of ANA, Rheumatoid Factor, CCP antibodies were repeated and turned out normal together with fecal calprotectin levels, CRP and ESR. In addition, the patients confirmed that they had no infections during the weeks prior to, or after, blood sampling. We therefore excluded infections as a cause of the high expression of inflammatory markers. Furthermore, when comparing the clinical parameters we found that the FM score in the “high inflammatory subset” showed a tendency to be slightly higher, but not significant, vs. the remaining 18 FM patients (FM-score 23.7 ± 6.0 vs. 21.2 ± 3.1, t-test = 0.17). On the other hand the duration of pain, the age, BMI, intensity of chronic abdominal pain, lower urinary tract symptoms, migraine, cognitive impairment, sick leave, number of drugs or fatigue severity score showed no differences between the two groups of FM-patients (FSS: 47.4 ± 5.0 vs 51.3 ± 9.9, t-test = 0.12).

### Increased Inflammatory Serum Protein Levels in FM Patients vs. Control Individuals

**Figure 3:**
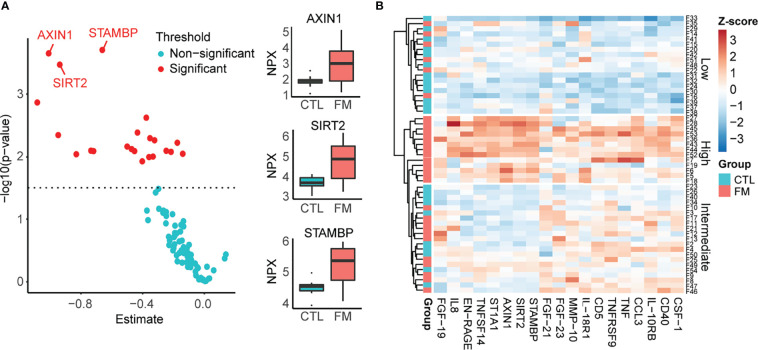
Relative levels of 92 soluble inflammatory proteins in sera of 30 FM patients vs. 23 matched control individuals, using Olink Inflammatory panel. **(A)** Graph showing relative increase of the 19 proteins (red spots) in the FM patient group. The most marked differences were identified for AXIN1, SIRT2 and STAMBP. **(B)** Heatmap showing increased levels of 19 inflammatory proteins in the group of FM patients. Individuals with low protein levels belong mainly to the control group (Low). A subset of patients shows a more pronounced increase in levels of inflammatory protein (High).

We then sought to complement our transcriptome analysis of B-cells by investigating the levels of soluble inflammatory mediators in our cohort of FM patients and controls. We investigated a panel of 92 inflammatory proteins (Olink Proteomics AB, Uppsala, Sweden) in sera obtained from 30 FM patients and 23 control individuals. Out of the 92 proteins, 19 showed increased levels in FM patients, whereas none showed reduced levels when compared to controls (**Figure 3**; **Tables 2**, **S5**). Among the inflammatory proteins, AXIN1, STAMBP and SIRT2 showed the highest relative increase in patients (**Figure 3A** **)**. A heatmap of the 19 inflammatory proteins revealed, similarly to the RNA sequencing data, a subset of patients (n=13) showing a more pronounced increase in inflammatory protein levels (i.e. patients F5, F6, F7, F18, F19, F27, F28, F36, F43, F44, F45, F52 and F53; **Figure 3B**). Furthermore, the clinical parameters of these 13 patients we found showed a significant higher FM-score (24.5 ± 4.0) when compared to the remaining 17 FM-patients (19.7 ± 2.7; p-value = 0.001). In addition, the 13 FM patients comprised five out of the seven patients in the “high inflammatory subset” defined from transcriptome profiling of B-cells, as well as eight out of ten patients enrolled from a Pain Clinic, representing a care setting managing cases with the most serious pain problems. However, no difference was found in fatigue severity score between the 13 patients and the remaining patients (52.5± 6.5 vs 48.7 ± 10.5, p-value = 0.27).

**Table 2:** Differential levels of inflammatory proteins.

Protein	UniProt	CTL	FM	Adjusted pval.
STAMBP	O95630	4.09	4.76	0.010
AXIN1	O15169	2.09	3.12	0.010
SIRT2	Q8IXJ6	3.43	4.39	0.010
ST1A1	P50225	3.21	4.32	0.031
MMP-10	P09238	9.51	9.96	0.044
FGF-23	Q9GZV9	1.64	1.83	0.044
IL-18R1	Q13478	8.82	9.17	0.044
TNFSF14	O43557	6.93	7.47	0.044
FGF-21	Q9NSA1	4.18	5.13	0.044
TNF	P01375	2.96	3.32	0.044
CD5	P06127	5.82	6.07	0.048
CD40	P25942	11.73	12.05	0.048
CCL3	P10147	6.53	6.99	0.048
IL-10RB	Q08334	6.54	6.76	0.048
FGF-19	O95750	8.78	9.51	0.048
TNFRSF9	Q07011	6.97	7.31	0.048
IL8	P10145	6.83	7.30	0.048
EN-RAGE	P80511	5.25	5.99	0.048
CSF-1	P09603	10.72	10.86	0.049

List of differentially expressed serum proteins levels between FM patients (FM; 30 patients) and controls (CTL; 23 healthy individuals), using Olink Inflammatory panel. Protein levels are expressed as normalized protein expression (NPX) values (log2 scale). Significant threshold is set as adjusted p-value < 0.05.

## Discussion

In the present study we used RNA-sequencing of B-cells complemented by the analysis of inflammatory proteins in sera obtained from FM patients and matched healthy control individuals. Our aim was to explore the role of B-cells in the pathophysiology of FM and to identify candidate soluble serum biomarkers for the disease. Analysis of the transcriptomes revealed an increased IFN-score in our group of FM patients, confirming that the disease is associated with an IFN signature in B-cells. Furthermore, we show that increased levels of a set of cytokines and chemokines in serum are associated with FM. Our results are in line with prior studies of an altered inflammatory profile in FM and we show for the first time the involvement of B-cells in the pathophysiology of the disease. The RNA-sequencing analysis uncovered a marked upregulation of a subset of genes associated with inflammation and autoimmunity, such as *S100A8* and *S100A9*, *VCAM*, *CD163* and *ANXA1*. The *S100A8* and *S100A9* genes encode for calgranulins that are components of the calprotectin complex. The two proteins play a prominent role in the regulation of inflammatory processes and immune responses, particularly in neutrophil chemotaxis and adhesion (43). Interestingly, increased calprotectin levels have previously been reported in saliva of patients with FM (44) as well as in sera of patients with different inflammatory diseases, such as rheumatoid arthritis, cystic fibrosis, inflammatory bowel disease, Crohn’s disease, giant cell arteritis, cystic fibrosis, Sjögren’s syndrome, systemic lupus erythematosus and progressive systemic sclerosis (45–47). The *VCAN* gene encodes for Versican, a member of the aggrecan/versican proteoglycan family of proteins. In addition to its role as a major component of the extracellular matrix, Versican participates in the inflammatory response induced by IFNs and the type I IFN receptor (IFNAR1) (48). The CD163 gene encodes for a scavenger receptor protein of the hemoglobin- haptoglobin complex and it is a potent biomarker of different inflammatory diseases (49). Soluble CD163 in urine is a novel and useful biomarker to follow the activity in lupus nephritis and it has emerged as a potential biomarker in systemic sclerosis (50, 51). The *ANXA1* gene encodes for Annexin A1, also known as lipocortin I, that acts as an effector in the glucocorticoid-mediated response (52, 53). Despite its well-known anti-inflammatory activity there is still some controversy on the precise modulatory role of Annexin A1 in inflammation (54). High levels of circulating anti-Annexin A1 antibodies and high serum levels of free Annexin A1 has been found in SLE (55). It has been shown that Annexin A1 regulates TLR-mediated IFN-β production, and mice depleted of Annexin A1 produce significantly less IFN-β (56). In our study, none of the FM patients received corticosteroid therapy as a possible explanation for the observed upregulation of *ANXA1* expression. However, it may be hypothesized that increased endogenous cortisol following chronic pain and stress in our cohort of FM patients may have influenced *ANXA1* expression.

Moreover, we noted that a subset of seven patients had a more pronounced upregulation of inflammatory and interferon-related genes within the entire group of FM. However, questionnaires and repeated investigation of the seven patients did not reveal any significant correlation with clinical parameters. The absent correlation may be explained by the limited size of our cohort. Other possible explanations are the dynamic expression pattern of inflammatory genes in B-cells that alternates between high and low activity over time, and heterogeneity in the pathophysiology of the disease due to different inflammatory and autoimmune mechanisms.

The inexplicable and robust inflammatory profile and the interferon signature in our group of FM patients is in line with findings in a prior study on peripheral blood mononuclear cells (PBMC) of FM patients. Moreover, the finding of an interferon signature in FM suggests inflammatory mechanisms in the pathogenesis of FM and may open up for biological drugs as a treatment option similar to that used in other diseases characterized by interferon dysregulation. However, further studies are now needed and in larger cohorts to validate our observations.

In addition to the gene expression analysis in B-cells we determined the levels of 92 inflammatory serum proteins in FM patients and controls. Among a set of 19 upregulated cytokines and chemokines, the levels of AXIN1, SIRT2 and STAMBP were found markedly increased in our cohort of FM patients. Notably, the observation is in agreement with the top upregulated proteins in a prior study of plasma proteins derived from an independent FM cohort (14). Furthermore, we also confirmed increased levels of TNF-α, IL-8, CD40 and TNFSF14 (LIGHT) from other studies (13, 17–21) together with TNFRSF9 (CD137). The increased levels of CD40, TNFSF14 (LIGHT) and TNFRSF9 (CD137), all of them members of the TNF superfamily, are intriguing. Interactions between ligands in the TNF superfamily (TNFSF) and TNF receptor superfamily of receptors (TNFRSF) provide co-stimulatory signals that control survival, proliferation, differentiation, and effector function of T cells. The TNSF member LIGHT has been shown to be overexpressed in various autoimmune and inflammatory disorders. LIGHT binds to the HVEM receptor expressed by T lymphocytes and induces the expression of interleukin-8 (57–60). Similarly, CD40 and its interaction with CD40L, are implicated in the pathogenesis of autoimmune diseases, particularly in SLE nephritis (61). Despite the complex and yet unclear roles of these proteins in the pathogenesis of FM, our findings show a strong overlap with those in an independent cohort of FM patients and bring further support for chronic systemic inflammation in the pathophysiology of FM.

We also found increased levels of the chemokines CCL3 and IL8 in FM patients. This is consistent with results from other studies that has demonstrated increased chemokines levels in serum and in cerebral spinal fluid of FM patients (14, 18–20). In addition to their role in the control and recruitment of effector leukocytes during infection, inflammation, tissue injury and tumour growth, chemokines are implicated in synaptic transmission and increase the sensitivity to pain by direct action on chemokine receptors expressed in peripheral nerves, the dorsal ganglia and in the spinal cord (62, 63). There are now emerging evidences suggesting that altered chemokine levels may account for the central sensitization in FM patients and in other chronic pain conditions (64, 65).

The correlation between levels of inflammatory markers and severity of symptoms in FM patients were more clearly reflected in the inflammatory serum protein levels than in transcriptome signatures of B lymphocytes. Thirteen patients showed a more pronounced increase of inflammatory serum proteins and this correlated to their increased FM-score when compared to the remaining group of 17 FM patients. Furthermore, the 13 FM patients comprised 5 out of the 7 patients showing a marked upregulation of inflammatory and interferon genes in B-cells, as well as 8 out of a total of 10 patients in the cohort recruited from a Pain Clinic, suggesting a more severe form of the disease. The incomplete overlap between the group with elevated inflammatory serum proteins and the “high inflammatory subset” identified by RNA sequencing of B-cells may be explained by the contribution of other cell types important for the inflammatory response or, by the suggested heterogeneity in the pathophysiology of the disease. Still, the correlation between FM and increased levels of a set of inflammatory serum proteins, as well as the correlation between FM severity score and very high levels of serum cytokines in a subset of patients, suggest mechanistic links and candidate biomarkers for the disease.

Concerning the biological mechanisms underlying chronical inflammation in FM there are many hypothesis. One is the inflammasome activation. The inflammasome is a multiprotein cytosolic complex that is located in macrophages, dendritic cells, and some other immune cells and represents a first line of defence of the innate immune response. It generates active caspase-1 and then convert the cytokine precursors pro-IL-1β and pro-IL-18 into mature and biologically active IL-1β and IL-18, that trigger a series of inflammatory responses and pyroptotic cell death (66). Some authors have shown that coenzyme Q10 deficiency induces activation of the inflammasome complex NLPRP3 in FM resulting in increasing serum levels of IL-1β and IL-18 (67). In our study we did not found any significant increase in serum levels of IL-1β and IL-18, as result of inflammasome activation. We could only detect increased levels of interleukin-18 receptor 1 (IL-18R1) that specifically binds IL18, and is essential for IL18 mediated signal transduction. However at RNA level, enrichment analysis revealed overexpression of inflammatory process in B lymphocytes in FM such as Endogenous TLR signalling activation and KRAS activation that both have shown the potential to trigger inflammasome activation in other diseases (66, 68). Probably chronic inflammation in FM has driven by many interacting inflammatory mechanisms that share intracellular molecular signalling.

Our study did not find a correlation between “fatigue” scored by FSS and inflammation or interferon score. The lack of correlation may partially be due to the difficulty in estimating the severity of fatigue and its high variability in FM patients. The relationship between INF score and fatigue is still debated in primary Sjögren’s syndrome and a tendency for a negative correlation has been shown in some studies (33, 34). Further studies are now required to identify mechanisms and molecular pathways underlying fatigue in FM. Interestingly, most of our patients experienced positive effects on fatigue from regular and low intense exercise. This is in line with prior reports showing that aerobic regular exercise improves fatigue as well as pain in FM (69).

Our combined results add several aspects to FM and its underlying causes. In particular, we bring further support for immune system in the pathophysiology of FM and we provide data supporting mechanisms reminiscent of that in autoimmune disease. We present the first transcriptome profiling of B lymphocytes in FM patients, showing that B-cells are implicated in the interferon signatures associated with the disease. Importantly, our study uncovered increased serum levels of a subset of cytokines and chemokines, in particular AXIN1, SIRT2 and STAMBP, confirming findings in a prior study of FM patients (14). In addition, the levels of inflammatory proteins correlated with FM severity score among patients. Further studies are now required to clarify the role of these proteins in the pathophysiology of FM and if they may serve as diagnostic markers for the disease.

### Limitations

The commercial protein panel used (Olink Inflammatory panel) does not contain the proteins corresponding to the genes with increased expression in B-cells from our analysis. Therefore, we could not confirm results from the RNA-seq analysis.

We did not perform any flow cytometry analysis of purified B lymphocytes. Further studies are needed to clarify if the co-stimulatory molecules, such as CD40 and TNFSF14, show increased levels in B cells of FM patients.

Our study is based on a homogeneous population of Swedish descent and of the same ethnicity as FM patients in a prior study (14). Therefore, we cannot claim that the findings from our cohort are representative for FM patients of other ethnicities. Further studies on different populations are now required to replicate our findings and to clarify if the candidate biomarkers identified are valid for FM in a more global perspective.

## Data Availability Statement

Count data and sample sheet is available from the authors via SciLife FigShare: doi.org/10.17044/scilifelab.19307021.

## Ethics Statement

The studies involving human participants were reviewed and approved by Regional ethic committee of Uppsala (2019-00144). The patients/participants provided their written informed consent to participate in this study.

## Author Contributions

SF conceived and designed the study. SF, KJ, and BK enrolled the patients. KG performed B- lymphocytes isolation and RNA extraction. JK, SF, and ND analyzed data and JK made the figures. SF, JK, and ND drafted the manuscript. All authors contributed to the article and approved the submitted version.

## Funding

The study was funded by grants from the Healthcare Board, Region of Uppsala, Sweden (FoU grant to SF) with contributions from the Swedish Fibromyalgia Association (to SF), the Swedish Research Council 2020-01947 (to ND), Uppsala University and Science for Life Laboratory.

## Conflict of Interest

The authors declare that the research was conducted in the absence of any commercial or financial relationships that could be construed as a potential conflict of interest.

## Publisher’s Note

All claims expressed in this article are solely those of the authors and do not necessarily represent those of their affiliated organizations, or those of the publisher, the editors and the reviewers. Any product that may be evaluated in this article, or claim that may be made by its manufacturer, is not guaranteed or endorsed by the publisher.
