# Supplementary material for: Inflammation and Interferon Signatures in Peripheral B-Lymphocytes and Sera of Individuals With Fibromyalgia
Source: Front Immunol. 2022 May 26;13:874490. doi: 10.3389/fimmu.2022.874490 (PMC9177944; doi:10.3389/fimmu.2022.874490)
Supplement: Supplementary file 1 [file Image_1.pdf]

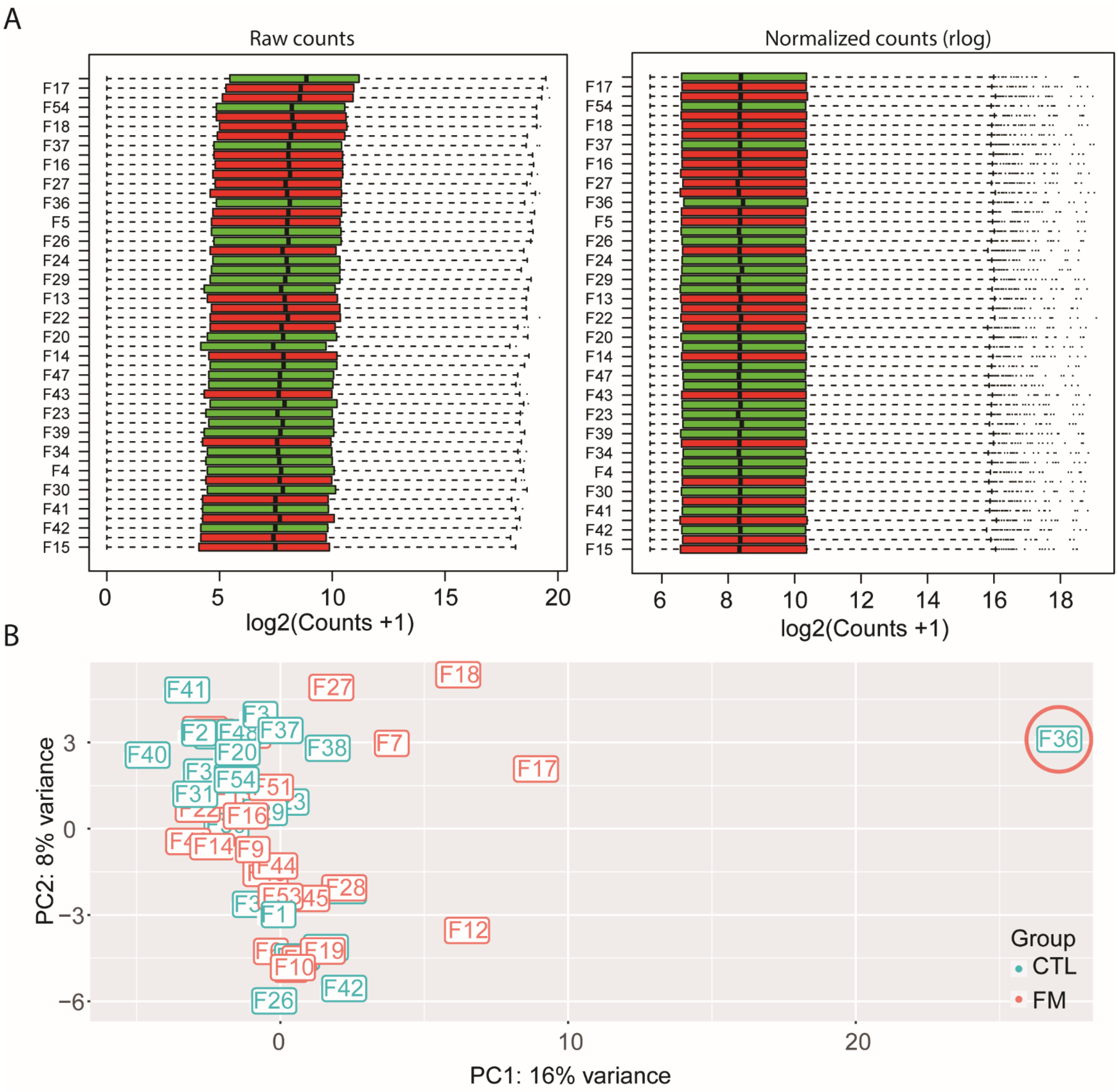

Figure S1. Quality assessment of the transcriptome data. A) Normalization of the raw counts (left) was applied in order to reduce the non-biologically derived variability generating normalized counts (right). B) Using a Principal Component Analysis (PCA) we identified one outlier sample (F36), which was removed in the further analysis.
