# Supplementary material for: Inflammation and Interferon Signatures in Peripheral B-Lymphocytes and Sera of Individuals With Fibromyalgia
Source: Front Immunol. 2022 May 26;13:874490. doi: 10.3389/fimmu.2022.874490 (PMC9177944; doi:10.3389/fimmu.2022.874490)
Supplement: Supplementary file 2 [file Image_2.pdf]

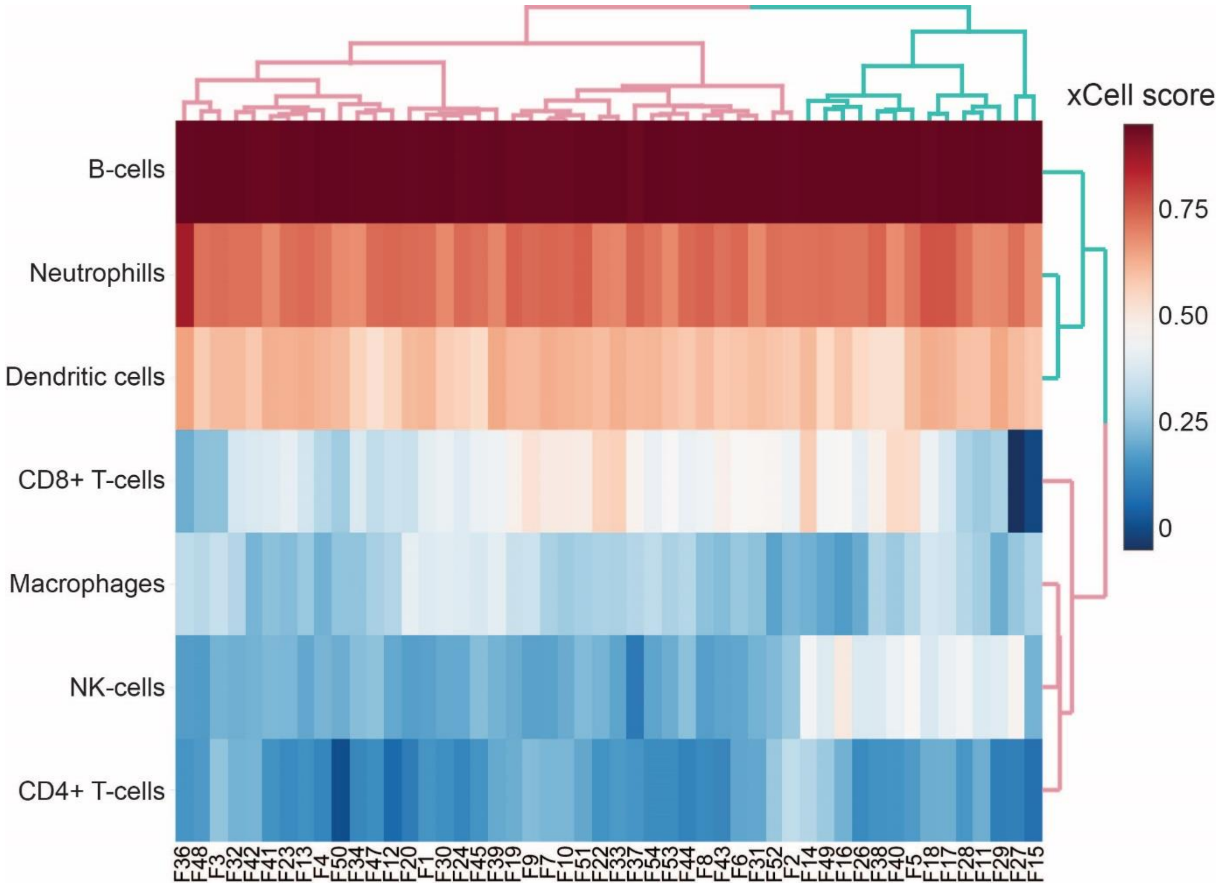

Supplementary Figure S2. Evaluation of cell signatures. Gene signature was evaluated using the xCell software. The cells are as expected mainly of a B-cell signature (average xCell-score 0.96 +/- 0.002). Secondary cell signatures include neutrophils (average xCell-score 0.73 +/- 0.03) and dendritic cells (DC; average xCell-score 0.61 +/- 0.03).
