# Supplementary material for: Inflammation and Interferon Signatures in Peripheral B-Lymphocytes and Sera of Individuals With Fibromyalgia
Source: Front Immunol. 2022 May 26;13:874490. doi: 10.3389/fimmu.2022.874490 (PMC9177944; doi:10.3389/fimmu.2022.874490)
Supplement: Supplementary file 3 [file Image_3.pdf]

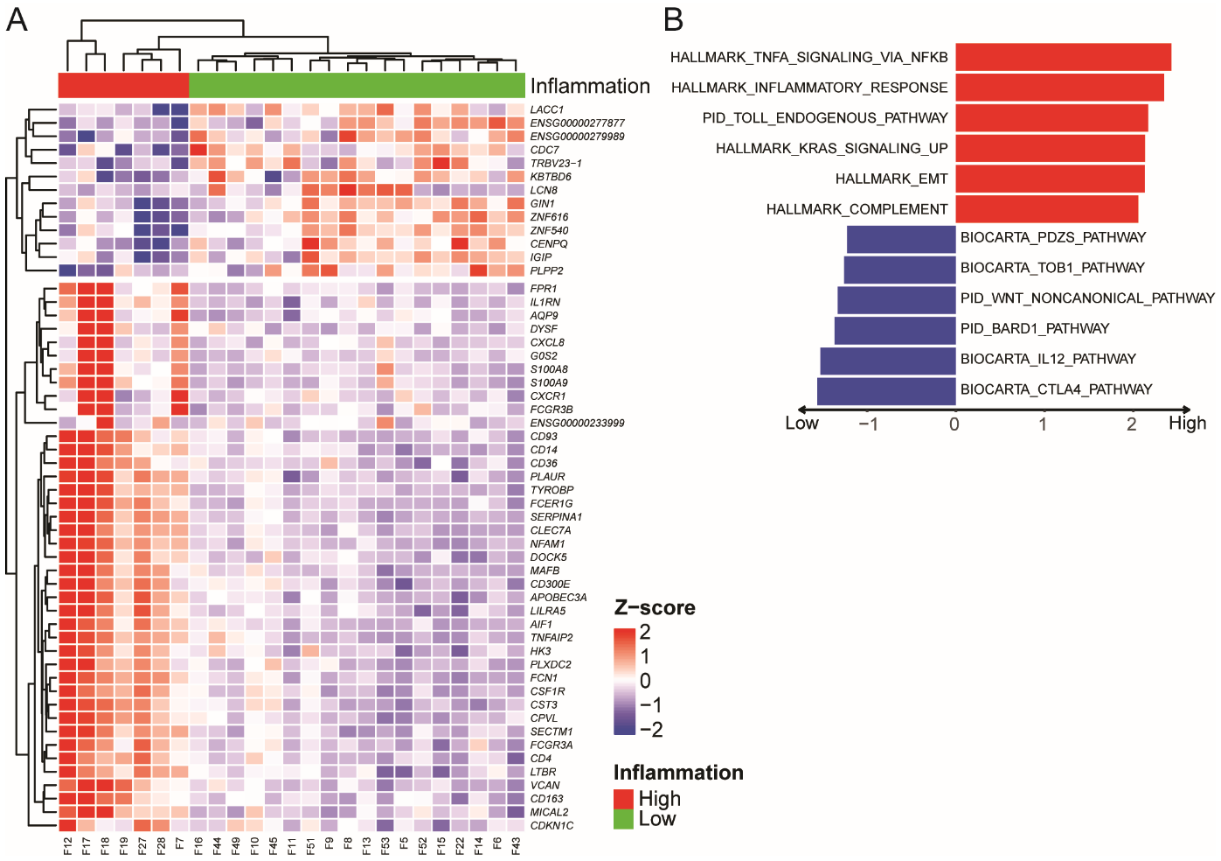

Supplementary Figure S3. Transcriptome analysis of the "high inflammatory" subset vs the other patients. A) Differential expression analysis confirm a higher expression of inflammatory genes in the high inflammatory subset of patients (7 patients) in comparison to the other patients (18 patients). B) The differentially expressed genes are mainly involved in TNF-α signaling and inflammatory response.
